# Exploring Effective Teacher-Student Interpersonal Interaction Strategies in English as a Foreign Language Listening and Speaking Class

**DOI:** 10.3389/fpsyg.2021.765496

**Published:** 2021-09-29

**Authors:** Jie Ding

**Affiliations:** Department of College English Studies, Luoyang Normal University, Luoyang, China

**Keywords:** interpersonal interaction skills/strategies, listening, speaking, EFL, L2 communication

## Abstract

The main purpose of English learning is to communicate and interact in global contexts. However, in English as a foreign language (EFL) contexts as in China, most of the students have limited interactional competence in contrast to their grammatical and structural competence. The reason is that Chinese classrooms mostly lack an interactional climate. This calls for an urgent need to develop interpersonal interaction skills by EFL teachers via appropriate strategies. To this end, this article presents an overview of nine interpersonal communication skills/strategies which are pivotal in L2 education. It also describes their definitions and related theories. Moreover, the outcomes of such strategies in aural skills are also explained. Finally, implications, research gaps, and future avenues for research are provided.

## Introduction

Over the past couple of decades, the educational context of China has made strident advancing steps regarding English language teaching and learning (Li et al., 2019). While China has the largest English as a foreign language (EFL) community, L2 students’ interactional skills lag behind their literacy skills (Zheng, 2019). They read and write well, but their aural skills (i.e., speaking and listening) are not as fluent as they should be. The reason for this deficiency is the classroom context and teacher-student interactions. The classroom environment is the focal venue for EFL students to develop their language competencies; hence, teachers are obliged to provide an interactive classroom setting that boots students’ meaning generation and negotiation. This calls for a shift of role on the part of the teachers in that they are no longer mere knowledge transmitters, but learning facilitators who set the scene for the development of interactional skills in their pupils (Littlewood, 2014). Another critical factor in determining L2 success interaction among teachers and students in the class. As natural and real-life encounters are usually scarce in EFL contexts, their burden is placed upon teachers and students to devise an interactional milieu that resembles and mirrors that of real life.

One of the most important tasks to be accomplished by L2 teachers and students in EFL settings, like China, is establishing a classroom climate based on positive interpersonal interaction skills. This necessitates a friendly rapport between the teacher and his/her students. Teachers must take into account the emotions and interests of their pupils in the class (MacIntyre et al., 2019; Wang et al., 2021). With this, they can create an interactive class where the students feel free to take part in L2 communication (Zheng, 2021). For a long time, this positive interpersonal kinship in the classroom has been confirmed to cause numerous positive outcomes in L2 education including achievement, well-being, engagement, learning, motivation, hope, and success, among others (Wendt and Courduff, 2018; Havik and Westergård, 2019). This approves that teaching is a relational job and the responsibility of learning is shared between the teacher and learners.

Now the intention of EFL classes is way beyond the simple presentation of linguistic information, but a place where social, psychological, and emotional interactions are provoked (Xie and Derakhshan, 2021). It is believed that an improved interpersonal interaction will create positive rapport in the class and removes negative stressors like distrust, doubt, anxiety, boredom, suspicion, and so forth. The reverse is also the case in which a positive learning environment generates interaction and communication among its parties. As pinpointed by Habash (2010), the quality of teaching in EFL contexts improves if teachers employ different interactional strategies that stimulate communication among learners. In their seminal papers, Gao (2021) and Xie and Derakhshan (2021) numerated nine key positive interpersonal communication skills/strategies including care, clarity, credibility, rapport with students, stroke, immediacy, confirmation, humor, and praise which will be explained later. Together with these, approximation, interpretability, discourse management, emotional expression, and interpersonal control are also known as effective interactional strategies (Gallois et al., 2005). Although many studies have been done on these strategies in different contexts pointing to their positive outcomes in academia, they have rarely been explored concerning aural skills in an EFL context. To fill this gap, the present mini-review article intended to define and explicate the concept of interpersonal communication/interaction, various strategies to improve it, related theories, and existing gaps and future trends in this line of inquiry.

## Literature Review

### The Concept of Interpersonal Communication/Interaction

Interpersonal communication which is at the core of the classroom process refers to communication that happens between people and forms a personal tie between them (Solomon and Theiss, 2013). In simple words, it is the exchange of information between two or more people sending and receiving the messages. Interpersonal communication is a unique kind of interaction among people as it highlights what occurs among them regardless of their place and presence (Wood, 2015). Going even further, McCornack (2010) and DeVito (2013) regarded the concept as a dynamic form of verbal and non-verbal interaction between people through which thoughts, behaviors, and emotions are conveyed. In the context of language teaching, interpersonal communication refers to the interaction (*verbal*, *non-verbal* = body language, and *para-verbal* = tone, intonation) that occurs among students and their teachers, which seeks to convey meaning, perform academic tasks, and establish relationships (Tranca and Neagoe, 2018). The type and degree of interaction in the classroom as a micro-social context varies across cultures, individuals, and subjects. Language education by nature calls for more interaction in comparison to hard sciences.

### Communication Accommodation Theory

As one of the most popular theories behind interpersonal interaction, Communication Accommodation Theory (CAT), grew out of Speech Accommodation Theory (SAT) proposed by Giles (1973). According to this theory, language speakers intentionally or unintentionally fine-tune their verbal and non-verbal behaviors to accommodate each other. This adjustment is done for arousing social approval, improving the effectiveness of communication, and maintaining a positive social identity (Beebe and Giles, 1984). The key strategies in CAT are *convergence* and *divergence* between interlocutors’ communication. People use convergence to adjust their communicative behaviors (verbal and non-verbal) to display similarity, get approval from the listener, or develop the conversation (Giles and Ogay, 2007). Convergence strategies include both verbal behaviors (e.g., repetition, praise, adjusting pronunciation, the speech rate, pause, the use of words and sentences, explanation, and even code-switching) and non-verbal behaviors (e.g., gestures, postures, smiling, and the like). On the contrary, divergence is a communication strategy employed by interlocutors to underscore the verbal or non-verbal difference between themselves and others. According to CAT, people manifest different social identities by using different language styles in terms of pronunciation, lexical differences, dialect, and other non-verbal behaviors. These strategies are of paramount importance in aural skills classes in that speaking and listening are quintessential samples of real-world communication where people aim to convey a message and understand it.

### The Attachment Theory

The attachment theory (AT) was proposed by British psychiatrist Bowlby (1969) to explain relational patterns among people. It maintains that human beings are born with an inherent need to establish a close emotional bond with others. It is at the core of children’s developmental psychology and maturation and argues that the attachment of a child to a caregiver produces a type of behavior that can later become autonomous (Zheng, 2021). Attachment is believed to be an emotional link among people which can influence their relationships, experiences, and task/work involvement. The theory is grounded on three main tenets including (1) bonding is an intrinsic human need, (2) the regulation of emotion and fear is done to increase vitality, and (3) attachment behaviors improve adaptiveness and development (Johnson, 2019). This theory is pertinent to L2 education in the sense that EFL students form emotional attachments with their instructors and peers. As posited in AT, EFL students who establish a strong affective attachment with their teachers have more tranquility to make discoveries and to socialize (Bergin and Bergin, 2009). Similarly, this emotional bond between students and the teacher increases EFL students’ courage and resilience when facing challenges. Hence, they are not afraid of failure and are intrinsically motivated and involved in classroom interactions (Bergin and Bergin, 2009). In a friendly environment as such, the level of classroom rapport is high and if a student does not know something, he/she will not freak out. In speaking and listening courses, teachers have to form secure attachments with their pupils to facilitate the ground for them to interact, cooperate, progress, and succeed. In EFL contexts, this sense of closeness is of utmost significance in that L2 education is full of linguistic, cultural, and social adversities that may preclude EFL learners’ interpersonal communication skills. Hence, this theory can pave the way for aural skills to develop in a democratic classroom climate.

### Nine Key Interpersonal Communication Skills/Strategies

Many interactional strategies may exist in English communication owing to its dynamic nature.

However, the most popular and well-documented ones suitable for EFL classes are those proposed by Gao (2021) and Xie and Derakhshan (2021) who proposed nine key positive interpersonal communication skills/strategies, namely *care, clarity, credibility, rapport with students, stroke, immediacy, confirmation, humor*, and *praise*. The concept of care was first proposed by Noddings (1984) to refer to a composite sense of compassion, openness to others’ needs, empathy in interactions, and closeness of a caregiver with a person as the receiver of the care. In the EFL context, teacher care concerns the delivery of genuine support to students, showing interest in their learning, and being empathetic toward them (Gabryś-Barker, 2016). The purpose of care is to fulfill students’ psycho-emotional needs via a positive and supportive environment (Laletas and Reupert, 2016). Next, clarity as an offshoot of a caring classroom refers to different strategies and approaches that EFL teachers and students utilize to ensure that their messages and ideas have been successfully understood by other parties (Bolkan, 2017). In interactive courses where students and teachers jointly construct the learning process, having clarity in talks and explanations is very important. Furthermore, credibility here means the degree of the believability of an interlocutor or the attitude of students toward their teachers regarding his/her competence, care, and trustworthiness (McCroskey and Young, 1981). Additionally, rapport is the harmonious teacher-student relationship characterized by joy, respect, and trust (Delos Reyes and Torio, 2020).

**FIGURE 1:**
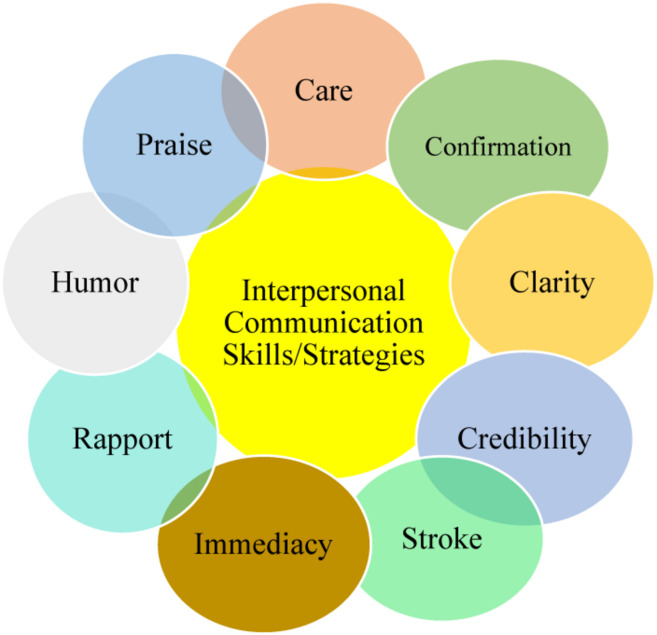
Interpersonal communication skills/strategies.

The concept of stroke concerns one’s natural expectation of recognition by others. In the EFL classroom, teachers are the strokers and learners are strokees. By nature, students search for teachers’ stroke or recognition and when it is absent, they feel deprived in the learning process (Xie and Derakhshan, 2021). As an interpersonal strategy highly related to rapport, immediacy concerns the proximity, closeness, and approachability of the teacher and students. In this case, their physical and effective distance is reduced. Moreover, confirmation refers to various communicative attempts to show students that they are valuable (Burns et al., 2017). It manifests itself in answering students’ questions and providing feedback, displaying passion in students’ learning, and involving in an interactive teaching style (Ellis, 2000). Students inherently seek teachers’ confirmation of their thoughts, feelings, and attempts in the class. Another interpersonal interaction strategy is humor which changes and enlightens the atmosphere of the class and affects its emotional climate making it appropriate for learning. Finally, teacher’s praise refers to the positive response to students’ behaviors and performances which go beyond simple feedbacks but confirmations or approval of learners’ efforts which increases their motivation and interaction in the class (Figure 1).

### The Application of Interactional Strategies to English as a Foreign Language Speaking and Listening

The mentioned nine fundamental interpersonal communication strategies and other linguistic strategies apply to speaking and listening skills in that they can encourage learners to cooperate, engage in the class, and form a positive rapport that facilitates interactions. In aural skills which are more demanding for EFL students, immediacy, care, and clarity put learners at ease and inspire them to speak out without fear of being wrong. Similarly, these interpersonal strategies allow learners to make interpretations about listening tasks in a friendly atmosphere that gives positive feedback. As aural skills are full of hesitation, pauses, informal phrases, hedges, and redundancies, EFL students usually feel dubious to initiate an interaction of making interpretations. Consequently, these positive interpersonal interaction strategies can soothe the tension and help learners decode and encode language with great panache. Moreover, these strategies generate motivation, enthusiasm, interest, and willingness to engage in speaking and listening activities.

In a global context, studies have been carried out on interactional strategies in the EFL setting, especially from the perspective of learners and teachers. Cohen et al. (1996) emulated the impact of strategies-based instruction on speaking a foreign language. As stated by Mufanti (2014), there exists a connection between low-level speaking proficiency and inappropriate social strategies. Bahadori and Tabatabaei (2016) investigated the effectiveness of interactional strategies on speaking accuracy and fluency of EFL learners based on gender. Astutik (2017) showed his concerns about lower learner’s interactional strategies in a public speaking class setting. Forbes and Fisher (2018) depicted the impact of students’ use of metacognitive learning strategies on confidence and proficiency in foreign language speaking skills.

The appropriate and purposed use of effective interactional strategies in teaching contributes to successful interaction and pedagogic qualities of competent teachers (Chiang, 2006; Garton, 2012; Astutik, 2016). Besides learners and instructors, the interface and media have been observed and revisited. The function of content and technologies in interaction is the newly discussed topic. The interaction between learners and peers is also concerned by scholars. Gómez–Rodríguez (2010) has analyzed how English textbooks help learners in EFL setting to develop their communicative competence. Trajtemberg and Yiakoumetti (2011) proposed web blogs as a tool for EFL interaction. York and Richardson (2012) explored the influential factors about interpersonal interaction in the online courses through a phenomenological study. Sun and Lee (2017) investigated the network-assisted learning environment of three countries’ EFL learners to show the compensation for the lack of linguistic competence with online interactional practice. Van Batenburg et al. (2019) was concerned with the prescribed interaction tasks on the textbook and learning material. Mehall (2020) explored the purposeful interpersonal interaction in the online course. Dao (2020) investigated the effect of interactional strategies instruction on learners’ engagement in peer reaction.

In EFL speaking or listening setting, effective engagement is the premise of interaction. It becomes the focus to motivate and guide low-level learners’ engagement through various communication strategies (Bejarano et al., 1997). Furthermore, scholars are also aware of the impact of cognitive factors, social environment, language policy, and media technology on EFL interaction. The concept of scaffolding in teacher-student interaction has received a great deal of attention in EFL educational research over the past few decades. Scaffolding is closely connected with the socio-cultural theory of Vygotsky. The effectiveness of interactional scaffolding has been examined and diagnosed. Scaffolding instruction intends to help learners maximize their needs and improve learners’ abilities to overcome the pre-problem and extend the scope of new competence in aid of various patterns of scaffolding. No matter in an authentic classroom setting or a network-based setting in EFL, interaction is a complicated but indispensable part of EFL speaking and listening class. There is an increasing tendency in class size, attendance number, the type of course in EFL speaking and listening setting. Are there essential distinctive features or real standards to examine and evaluate the interaction degree in regard to the effect, the acceptance and the prospect? The learners’ personal feeling of the perception of different interaction strategies is real and full of emotion. As a case study, it is characteristic and referential. The analysis of interactional strategies effects data and structure types helps to describe and analyze the research object more objectively.

The current teaching technologies and social demands of language learning provide learners or instructors to reexamine and reflect the relationship between learners and instructors. With the aid of current various multi-media, EFL interaction typologies extend to learner-learner, learner-instructor, and learner-media-instructor style. Especially due to the severe spread of the COVID-19 pandemic in the global area, the flipped classroom or online teaching and learning becomes essential and presents a new tendency of knowledge inquiry and interpersonal interaction. Even though online teaching endows participants with more freedom and flexibility, it challenges effective interpersonal interaction. The purposed interpersonal interaction strategies illustrate the new technology-self and information-self to build a new balanced and rapport relationship to gain knowledge. Effective interpersonal communication skills are impellers of EFL practices. It seems new media and technologies provide other interaction patterns, besides learners-learners and learners-instructors. The learning materials and assistant technology have become part of the media of interaction. Thereby, another interactional pattern emerges, learner-media-instructor style, especially in an AI intelligent age and mask-wearing social interactions. Online learning or interaction in EFL becomes appropriate and necessary currently.

### Implications, Research Gaps, and Future Directions

In light of this review article, it can be argued that the use of effective interpersonal communication skills/strategies has implications for EFL teachers, students, teacher educators, and researchers. From the EFL teaching evaluation level, the judgment and evaluation of effective interactional strategies in the EFL setting lead to further reflection and exploration about the process of teaching and learning agents, processes and effects. The nine communicative strategies just provide more facets to conceptualize the effect of the interactional practice. EFL teachers can use the results to create a positive classroom climate in which these nine strategies are employed in the class, especially in aural skills courses. Moreover, their knowledge of the emotional aspects of L2 education will increase. Likewise, EFL students can play their part in forming a positive rapport in the class that encompasses the rest of interpersonal skills. From the teaching training or self-development aspect, teacher educators can run workshops and training courses on teaching interpersonal interaction strategies to pre-service and in-service EFL teachers offering practical techniques regarding each skill. L2 researchers can use the idea proposed in this article and run cross-cultural studies to see if such strategies vary across different level learners or contexts. From the research content and methods, there are various meaningful perspectives to be mentioned and focused on in learning and instruction of EFL settings. The emotional effect of interactional strategies is worthwhile to be further discussed, especially in the flipped or blending EFL setting. Due to the appearance of the new interactional facilities, personal communicative software and interactional platforms extend the manner and the content of the interaction. The compensation and scaffolding function of interactional strategies in media-assisted EFL settings extend the scope and content of effective teacher-student interaction. Most studies on these constructs have been quantitative and one-shot; hence, scholars can conduct qualitative, mixed-methods, and longitudinal studies. EFL teachers’ and students’ perceptions and practices of these strategies are also recommended to future researchers. The comparative studies and field researches of intercultural communication in EFL online learning about different learners, contents, and manners can be further explored. Finally, experimental studies can be done on teaching these nine interpersonal interaction skills in China and other contexts to see if they can be improved by appropriate intervention.

## Author Contributions

The author confirms being the sole contributor of this work and has approved it for publication.

## Conflict of Interest

The author declares that the research was conducted in the absence of any commercial or financial relationships that could be construed as a potential conflict of interest.

## Publisher’s Note

All claims expressed in this article are solely those of the authors and do not necessarily represent those of their affiliated organizations, or those of the publisher, the editors and the reviewers. Any product that may be evaluated in this article, or claim that may be made by its manufacturer, is not guaranteed or endorsed by the publisher.
